# Differential effects of typical and atypical antipsychotics on cognitive function in schizophrenia

**DOI:** 10.1192/j.eurpsy.2026.12145

**Published:** 2026-07-31

**Authors:** D. Njah, M. Lagha, K. Maagli, A. Zyed, W. Homri

**Affiliations:** Psychiatry c, Razi psychiatric hospital, Manouba, Tunisia

## Abstract

**Introduction:**

Antipsychotics are a mainstay of schizophrenia treatment, effectively controlling psychotic symptoms. However, their impact on cognitive function is attracting growing interest, as cognitive impairment is a major factor in functional disability, affecting memory, attention, and executive functions and consequently impairing quality of life. Understanding the effect of different types of antipsychotics on cognition is essential for adapting therapeutic strategies and optimizing patients’ daily functioning.

**Objectives:**

Our objective was to assess the effect of typical and atypical antipsychotics on cognitive function in patients with schizophrenia and schizoaffective disorder (SAD).

**Methods:**

This was a cross-sectional study including patients with schizophrenia and SAD, aged between 18 and 65 years, that took place over a 2-month period, from February 1, 2025 to April 15, 2025 who were followed up at RAZI psychiatric Hospital in Tunisia. Cognitive function was assessed using the Arabic version of the Montreal Cognitive Assessment (MoCA) scale. Details of patients’ antipsychotic prescriptions were obtained through review of their medical files.

**Results:**

We included 56 patients with schizophrenia and 14 patients with SAD with a mean age of 42 years (SD, 11). Sixty seven percent of our population were male(n=47) and almost 76% (n=53) were single. Among the patients, 27% (n = 19) were receiving atypical antipsychotics (AAP), 52% (n = 36) were on classical antipsychotics (CAP), and 21% (n = 15) were prescribed a combination of both. The median MoCA score was 20(interquartile range, 16-25; range 5-30). Cognitive dysfunction was found in 74,3% of our population(n=52). Patients receiving AAP had significantly higher MoCA scores (p = 0.005), whereas those on CAP showed significantly lower MoCA scores (p = 0.001).

**Conclusions:**

Our findings suggest that the use of first-generation antipsychotics is associated with cognitive impairment, whereas atypical antipsychotics exert a protective effect. These results highlight the importance of carefully selecting antipsychotic treatment to optimize cognitive outcomes in patients with schizophrenia and schizoaffective disorder.

**Disclosure of Interest:**

None Declared

